# Editorial: Transcriptional and epigenetic regulation in normal and aberrant hematopoiesis

**DOI:** 10.3389/fmolb.2024.1388983

**Published:** 2024-03-13

**Authors:** Jonas S. Jutzi, Ruud Delwel, Robert Zeiser, Anna E. Marneth

**Affiliations:** ^1^ Division of Hematology, Department of Medicine, Brigham and Women’s Hospital, Harvard Medical School, Boston, MA, United States; ^2^ Department of Hematology, Erasmus MC Cancer Institute, Rotterdam, Netherlands; ^3^ Department of Hematology, Oncology, and Stem Cell Transplantation, University Medical Center Freiburg, Freiburg, Germany; ^4^ CIBSS Centre for Integrative Biological Signaling Studies, Freiburg, Germany; ^5^ Department of Laboratory Medicine, Laboratory of Hematology, Radboud University Medical Center, Nijmegen, Netherlands

**Keywords:** hematopoiesis, myeloid malignancies, transcriptional regulation, epigenetics (chromatin remodelling), epigenetics (DNA methylation)

Hematopoiesis constitutes a process in which multipotent progenitors undergo differentiation, proliferation, cell-cycle arrest, or apoptosis. This tightly balanced process is governed by epigenetic modifications resulting in changes in gene expression. Although recent developments in sequencing technologies, chromatin structure analyses, and bioinformatic tools have increased our knowledge of gene regulation, our understanding of (epi)genetic regulation in normal and aberrant hematopoiesis remains incomplete.

The aims of this Research Topic “T*ranscriptional and Epigenetic Regulation in Normal and Aberrant Hematopoiesis*” are (**1**) to review the epigenetic landscape and transcriptional control of normal and aberrant hematopoiesis, (**2**) to cover timely and novel discoveries in both, fundamental and translational research focused on epigenetic modifiers, transcription factors, RNA splicing factors, or new developments on related proteins. This Research Topic in *Frontiers in Biomolecular Biosciences* contains four intriguing publications that **(1)** clarify our current understanding of blood cell development as well as leukemia induction and progression in humans using various sequencing-based technologies, **(2)** give insight into epigenetic regulation of normal and malignant hematopoiesis, **(3)** discuss the most novel insights into DNA methylation at the single-cell level, and **(4)** summarize the consequences of cohesion mutations in myeloid malignancies.

The development of next-generation sequencing methods has revolutionized our understanding of normal and aberrant hematopoiesis with regards to mutation calling, key regulatory pathways, gene expression profiles and epigenetic patterns. To this end, Heuts and Martens have reviewed sequencing-based technologies and human cell systems used for the understanding of blood cell development and leukemia. They expand on the role of transcription factors and enhancers, non-coding DNA sequences, and the methodologies developed to study gene expression regulation. They convey the latest findings from studies using innovative combinatory single-cell RNA sequencing and single-cell mutation calling techniques in normal and malignant hematopoiesis. Moreover, the authors discuss how induced pluripotent stem cells can be used to deepen our understanding of normal and malignant hematopoiesis. Finally, they highlight the enormous impact of genomics and transcriptomics data in the prognostication of patients with hematological malignancies and how such data have enabled the development of novel therapies.

The second paper on our Research Topic, by Wallace and Obeng, is entitled “Wallace et al.” Our negatively charged DNA is wrapped around a core of positively charged histone proteins. Chemical modifications of DNA or histones can alter the accessibility of genomic regions and chromatin structure, which is one of the many ways in which gene expression is regulated. Additionally, RNA modifications and their binding proteins regulate isoform expression and transcript stability, providing another layer of gene expression regulation. These so-called epigenetic regulation mechanisms occur independent of changes in DNA sequences. Over the past decades, it has become clear that many epigenetic regulators are mutated in hematological malignancies and might be viable targets for therapeutic intervention. Wallace and Obeng provide an overview of epigenetic regulators and consequences of mutations in those genes in hematological malignancies, and potential targets for clinical drug development.

Thirdly, Albinati et al. contributed an opinion piece on the emerging field of opportunities for **single-cell DNA methylation** studies. A high level of promoter DNA methylation has typically been associated with repression of gene expression, while a low level of promoter DNA methylation has been correlated with genes that are highly expressed. However, DNA methylation patterns are much more complex and can be used for other purposes than correlation with expressed or non-expressed genes. Indeed, Albinati et al. provide evidence that studying DNA methylation in enhancer regions provides information on cell proliferation and may be used to determine the **cellular origin of tumors**. Furthermore, they discuss challenges and opportunities for single-cell DNA methylation analyses. In this section, they delve into the state-of-the-art techniques available for studying DNA methylation, their resolution, and consider associated costs. Based on this, they provide recommendations as to the scenarios in which these techniques are most useful.

Finally, going into specific consequences of mutations in an epigenetic regulator in myeloid malignancies, Bhattacharya et al. summarizes the clinical correlations and molecular consequences of mutations in the cohesion complex member *STAG2*. The cohesion complex is a ring-like structure important for sister chromatid cohesion and promoter-enhancer looping in cells, thereby playing essential roles in cell division, DNA repair, maintenance of genomic integrity, and transcriptional regulation. Bhattacharya et al. highlight that disruptive *STAG2* mutations correlate with dismal prognosis in both hematological malignancies as well as solid tumors. Moreover, *STAG2* mutations cause increased hematopoietic stem and progenitor cell self-renewal and impaired myeloid differentiation. Although STAG2 can be replaced by its homologue STAG1 in the cohesion complex, genomic binding studies show that STAG1 cannot fully compensate for STAG2 loss. The authors discuss various models of co-mutation of STAG2 with other genes implicated in myeloid malignancies, and how the order of acquisition of mutations can lead to different phenotypes. Finally, gene programs regulated by STAG2 and potential therapeutic approaches preferentially targeting *STAG2* mutated cells are discussed.

Together, this Research Topic provides an overview of the most recently applied technologies and findings in transcriptional and epigenetic regulation of hematopoiesis. These include tremendous advancements in next-generation sequencing methodologies and bio-informatic pipelines that have been realized over the past decade. This resulted in the implementation of these technologies in clinical diagnostics. It further resulted in a deep molecular understanding of hematological malignancies regarding their genetic make-up and aberrant regulation of gene expression. Subsequently, this has enabled researchers to develop therapies targeting mutant proteins and identify molecular vulnerabilities, improving prognostication and treatment of hematological malignancies. We are hopeful that by using such molecular insights, we will eventually develop personalized therapeutic strategies to better treat and eradicate these life-threatening diseases.

